# White Matter Abnormalities in Patients with Focal Cortical Dysplasia Revealed by Diffusion Tensor Imaging Analysis in a Voxelwise Approach

**DOI:** 10.3389/fneur.2012.00121

**Published:** 2012-07-26

**Authors:** Viviane de Carvalho Fonseca, Clarissa Lin Yasuda, Guilherme Garlipp Tedeschi, Luiz Eduardo Betting, Fernando Cendes

**Affiliations:** ^1^Neuroimaging Laboratory, Department of Neurology, University of CampinasCampinas, Brazil

**Keywords:** extratemporal focal epilepsy, FCD, WM, DTI, FA, tract-based spatial statistic

## Abstract

**Background:** Diffusion tensor imaging (DTI) allows the analysis of changes in microstructure, through the quantification of the spread and direction of water molecules in tissues. We used fractional anisotropy (FA) maps to compare the integrity of WM between patients and controls. The objective of the present study was to investigate WM abnormalities in patients with frontal lobe epilepsy secondary to focal cortical dysplasia (FCD). **Materials and Methods:** We included 31 controls (12 women, 33.1 ± 9.6 years, mean ± SD) and 22 patients (11 women, 30.4 ± 10.0 years), recruited from our outpatient clinic. They had clinical and EEG diagnosis of frontal lobe epilepsy, secondary to FCD detected on MRI. Patients and controls underwent 3T MRI, including the DTI sequence, obtained in 32 directions and *b* value of 1000 s/mm^2^. To process the DTI we used the following softwares: MRIcroN and FSL/TBSS (tract-based spatial statistics). We used a threshold-free cluster enhancement with significance at *p* < 0.05, fully corrected for multiple comparisons across space. **Results:** Areas with FA reduction in patients were identified in both hemispheres, mainly in the frontal lobes, cingulum, and forceps minor (*p* = 0.014), caudate e anterior thalamic radiation (*p* = 0.034), superior longitudinal fasciculus (*p* = 0.044), uncinate fasciculus, and inferior fronto-occipital fasciculus (*p* = 0.042). **Conclusion:** Our results showed a widespread pattern of WM microstructural abnormalities extending beyond the main lesion seen on MRI (frontal lobe), which may be related to frequent seizures or to the extent of MRI-invisible portion of FCD.

## Introduction

Epilepsy secondary to focal cortical dysplasia (FCD) usually begins early in life, is often refractory to antiepileptic drug (AED) therapy, and a frequent cause of focal motor status or focal epilepsy, which may be life-threatening (Desbiens et al., 1993). The term FCD designates a spectrum of abnormalities of the laminar structure of the cortex (Guerrini and Parrini, 2010; Blümcke et al., 2011) and is present in a significant proportion of epilepsy surgical series, in particular in the pediatric population (Fauser et al., 2006; Blümcke et al., 2011).

Barkovich et al. (2001) classified different types of FCD as malformations due to abnormal neuronal proliferation or due to abnormal cortical organization, that may be located in areas of eloquent cortex. In addition, the epileptogenic zone may be more extensive than the lesion visualized on MRI alone (Palmini et al., 1995; Rosenow et al., 1998). Intracranial EEG recordings have demonstrated that epileptic activity exhibits complex propagation patterns within and between hemispheres (Duchowny et al., 2000), often extending beyond the MRI visible lesion (Tassi et al., 2002; Najm et al., 2007).

Diffusion tensor imaging (DTI) is a non-invasive imaging technique that can examine molecular diffusion of water within the brain by applying gradients in at least six non-collinear directions. DTI can evaluate white matter integrity and may detect abnormalities in earlier stages than conventional T2- or T1-weighted imaging (Pierpaoli et al., 1996; Lee et al., 2004). The measurement of white matter integrity used in this study is fractional anisotropy (FA), which is determined by the directional magnitude of water diffusion in three-dimensional space. The tract-based spatial statistic (TBSS) provides an automated whole brain voxel-by-voxel analysis of FA without *a priori* bias for different brain regions.

The aim of this study was to investigate white matter changes in patients with frontal lobe epilepsy due to FCD using DTI.

## Materials and Methods

We studied 31 controls (12 women, 33.1 ± 9.6 years, mean ± SD) and 22 patients (11 women, 30.4 ± 10.0 years) with MRI defined FCD, recruited from our outpatient clinic at our University Hospital from May 2009 to April 2010. Informed consent was obtained for participation in the studies, approved by the Internal Review Board of our institution.

Patients started seizures at mean age of 8.5 years and presented an average of 12.6 seizures per month. They had clinical and EEG diagnosis of frontal lobe epilepsy, probably secondary to FCD (Table 1). Patients and controls underwent 3T MRI, including the DTI sequence. A standardized protocol was performed on a 3 Tesla Achieva-Intera Philips^®^, release 2.6.1.0. The DTI acquisition was performed in axial plane obtained in 32 directions; 2 mm thickness, with echo time 60, factor-*b* 1000, reconstructed matrix 128 × 127, field of view (FOV), 232 × 232. In addition, all patients had a 3T MRI epilepsy protocol that included 3 mm coronal T1-weighted inversion recovery, 3 mm coronal T2-weighted multi-echo sequence, 3 mm coronal and axial FLAIR, volumetric T1-weighted sequence with 1 mm isotropic voxels, and volumetric T2-weighted sequence with 1 mm × 1 mm × 1.5 mm voxels. All images were analyzed in a workstation with high resolution widescreen monitor. Multiplanar reconstructions on T1 and T2 volumetric sequences were performed for all patients. The neuroimaging characteristics for diagnosing probable FCD were based on a recent proposal for classification by Blümcke et al. (2011) and included increased cortical thickness, blurring of the cortical-white matter junction, increased signal on T2-weighted images, a radially oriented linear or conical transmantle stripe of T2 hyperintensity, cortical thinning, and localized brain atrophy (Blümcke et al., 2011).

**Table 1:** **Summary of clinical presentation of patients with focal epilepsy secondary to MRI defined focal cortical dysplasia**.

Patient	Age (years)	Sex	Age Sz. onset	Sz. Freq.	Family hist. Sz.	Sz. type	AEDs	FCD in MRI	EEG
1	27	F	13	6	Y	SPS-CPS-SGTC	TPM. LGT. CBZ	R frontal	L > R generalized ED
2	28	F	9	4	N	SPS-CPS	CBZ. TPM. CLB	L frontal	L frontal ED
3	17	F	2	4	Y	SPS-CPS	CBZ. VPA. TPM. CLB	L frontal	L temporal ED
4	28	F	10	8	N	SPS-CPS	CBZ CLB	R parietal frontal	Bilateral L > R ED
5	27	M	2	30	N	SPS-CPS-SGTC	DPK. LGT	R frontal	Bilateral synchrony ED
6	26	F	6	4	N	SPS-CPS	TPM. CBZ. C LB	R frontal	Bi-frontal ED
7	18	F	8	6	N	SPS-CPS	LGT. TPM. CLB	R frontal	R frontal ED
8	17	M	0.6	60	Y	CPS-SGTS	TPM. DPK. CLB	R frontal	R frontal ED
9	39	M	25	1	N	CPS-SGTS	CBZ	L frontal	L frontal ED
10	32	F	7	4	N	SPS-CPS-SGTC	CBZ. TPM. CLB	L frontal	L frontal ED
11	36	F	2	1	N	CPS-SGTS	LGT. CLZ	R frontal	R frontal ED
12	49	F	13	1	N	CPS-SGTS	OXC	L frontal	Bi-frontal ED
13	33	M	4	4	Y	CPS-SGTS	LGT. VPA. CLZ	R frontal	R frontal ED
14	29	M	5	0.5	N	SPS-CPS-SGTC	CBZ	R frontal	R > L generalized ED
15	26	M	17	2	N	SPS-CPS	CBZ. CTG. CLB.	R temporo-frontal	Bi-temporal ED
16	23	F	9	12	Y	SPS-CPS-SGTC	LGT. CLB	L frontal	L frontal ED
17	59	F	18	20	N	SPS-CPS	CBZ	L frontal	L frontal ED
18	30	M	13	0.5	Y	CPS-SGTS	CBZ. LGT. CLB	L frontal	L > R generalized ED
19	18	M	0.8	30	Y	SPS-CPS	PB. DPK	R frontal	Bi-frontal ED
20	34	M	7	3	Y	CPS-SGTS	CBZ. TPM	L frontal	L frontal ED
21	37	M	6	16	N	SPS-CPS	CBZ. VPA	R temporo-frontal	R frontal ED
22	36	M	9	60	Y	SPS-CPS-SGTC	CBZ. CLB. TPM	L frontal	L frontal ED

*Age Sz. onset, age at seizure onset in years; family hist. Sz., presence (Y) or absence (N) of family history of seizures; Sz. type, type of seizures; SPS, simple partial seizures; CPS, complex partial seizures; SGTC, secondary generalized tonic-clonic seizures; AEDs, antiepileptic drugs; TPM, topiramate; LGT, lamotrigine; CBZ, carbamazepine; CLZ, clonazepam; DPK, divalproate; VPA, valproic acid; CLB, clobazan; OXC, oxacarbazepine; PB, phenobarbital; FCD in MRI, localization of focal cortical dysplasia on magnetic resonance imaging; EEG, electroencephalography; R, right; L, left; ED, epileptiform discharges*.

All patients were under investigation for epilepsy surgery and Table 1 shows the summary of their investigation. At this point only four of these patients have confirmation of FCD by pathology.

For processing the DTI, all imaging data were transferred to a cluster of Linux workstations. The structural images were visually inspected for any structural abnormalities by a neuroimaging expert. First DICOM images were converted to 4D-Nifti file using the MRIcroN program. Preprocessing and analyses of diffusion data was done with an in-house protocol using FSL. For voxelwise analysis of FA values we applied TBSS (also included in FSL), with threshold-free cluster enhancement with significance at *p* < 0.05, fully corrected for multiple comparisons across space (Smith et al., 2006). For localizing the significant results we used the atlases offered by FSL (http://www.fmrib.ox.ac.uk/fsl/data/atlas-descriptions.html#wm).

Of all patients included in the study, 12 had right-sided lesions and 10 had left-sided MRI lesions (male *n* = 11; female *n* = 11).

The patients composed three groups for analysis. First the control group was compared with patients group who had right-sided FCD. The second analysis compared the control group with those patients that had left-sided FCD. Next, we analyzed all patients after flipping the images with right-sided lesions to the left side, versus controls.

## Results

**Figure 1:**
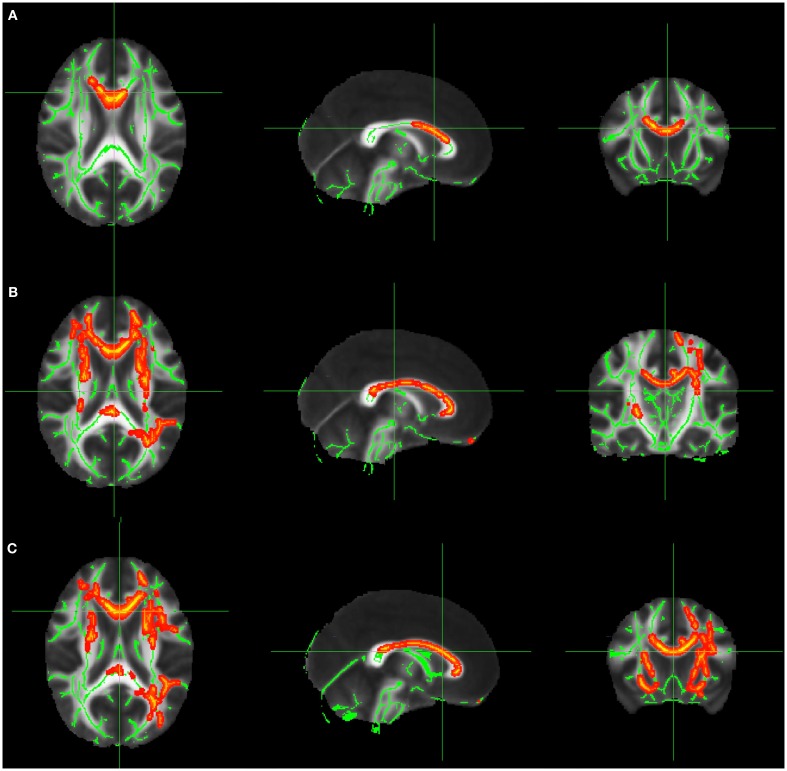
**(A)** FA decrease in patient group with right-sided FCD. **(B)** FA decrease in patient group with left-sided FCD. **(C)** FA decrease in the analysis of patients with left and right-sided lesions altogether after flipping the MRIs with right-sided FCD lesions. The yellow/red voxel indicated brain regions where the FA was significantly reduced in patients with focal epilepsy when compared to controls.

1. Comparison of FA values in patients with right-sided lesions versus normal controls. We identified a reduction of FA in these patients mainly in: forceps minor (*p* = 0.032), ipsilateral hemisphere, forceps minor (*p* = 0.042), and cingulum (*p* = 0.048) in the contralateral hemisphere (Figure 1A; Table A1 in Appendix).
2. Comparison of FA values in patients with left-sided lesions versus normal controls. Tract-based spatial statistic analysis showed extensive reduction of FA in areas involving both hemispheres; right anterior thalamic radiation and forceps minor (*p* = 0.022), left corticospinal tract and right corticospinal (*p* = 0.022), right inferior fronto-occipital fasciculus (*p* = 0.022), left cingulum e forceps minor (*p* = 0.014), anterior thalamic radiation, fasciculus uncinatus, left inferior fronto-occipital fasciculus (*p* = 0.036), left superior and inferior longitudinal fasciculus, and forceps major (*p* = 0.034; Figure 1B; Table A2 in Appendix).
3. Comparison of FA values in all patients after flipping the images with right-sided lesions to the left side versus normal controls.

In this analysis the MRIs of patients with right-sided lesions were flipped, so all the lesions would be on the same side. We identified areas with reduced FA in the left superior longitudinal fasciculus (*p* = 0.022), left inferior longitudinal fasciculus (*p* = 0.032), inferior fronto-occipital fasciculus (*p* = 0.038), right and left anterior thalamic radiation (*p* = 0.042 and 0.022, respectively), left corticospinal tract (*p* = 0.042), cingulum and left forceps major (*p* = 0.048), left inferior fronto-occipital fasciculus (*p* = 0.032; Figure 1C; Table A3 in Appendix).

## Discussion

We found a widespread pattern of WM microstructural abnormalities extending beyond the MRI visible lesion and putative epileptogenic area in patients with frontal lobe epilepsy secondary to MRI identified FCD.

Both groups (left and right-sided MRI lesions) demonstrated bilateral and widespread FA changes; however, these were slightly more extensive in the group with left-sided FCD. Perhaps this could be related to the fact that the dominant hemisphere for language may be more vulnerable to damage.

In one study (Eriksson et al., 2001) of 22 epilepsy patients with FCD (although only four had MRI evidence of isolated lesions), all patients had DTI abnormalities corresponding to the MRI lesion. Fifteen of these patients had areas of structural disorganization as shown by decreased anisotropy or increased diffusivity values outside the lesion visible on conventional MRI (Eriksson et al., 2001).

Decrease of FA was also detected in the superior longitudinal fasciculus in both analyses. This is the largest fiber tract of the long association fiber system and connects the prefrontal, parietal, and temporal cortices (Catani et al., 2002). We also found FA reduction in the uncinate fasciculus, which connects the anterior temporal lobe with medial and orbital prefrontal cortex areas in a bidirectional way (Schmahmann and Pandya, 2006).

Reduced FA may indicate one of the three processes (or a combination of these): degradation of both axonal membranes and myelin (Beaulieu et al., 1996; Pierpaoli et al., 2001), abnormalities of myelin with sparing of the axons (Gulani et al., 2001; Song et al., 2002), or reduced density of myelinated axons (Takahashi et al., 2002).

Unfortunately, we were not able to perform correlations between EEG abnormalities and FA abnormalities, or between the extent of visible FCD and the degree of FA abnormalities, because this was a group analysis. Another limitation of this paper is that we had confirmation of FCD on histopathology in only four patients, since the others are still waiting or refused surgery.

Diffusion tensor imaging and tractography in patients with FCD may be useful in the detection and delineation of MRI-invisible structural abnormalities, and in determining both the connectivity of a given area of the cortex and the spatial relationship between the lesion and major white matter tracts.

Further studies need to be conducted to evaluate the usefulness of DTI for the delineation of white matter abnormalities in patients with epilepsy.

## Conflict of Interest Statement

The authors declare that the research was conducted in the absence of any commercial or financial relationships that could be construed as a potential conflict of interest.

## Appendix

**Table A1:** **Areas of significant reduction of FA values for the comparison of patients with right-sided focal cortical dysplasia lesions versus normal controls**.

Fractional anisotropy decrease – FCD right-sided					
	JHU		MNI	*X, Y, Z*	*p*-Value
3%	R forceps minor	8%	Frontal lobe	83, 145, 91	0.032
3%	L forceps minor	6%	Frontal lobe	93, 143, 91	0.042
5%	Forceps minor	1%	Frontal lobe	99, 148, 91	0.048
8%	Cimgulum	1%	Frontal lobe	99, 148, 91	0.048

*JHU, Areas according to the JHU white matter atlas; MNI, Areas according to the MNI coordinates*.

**Table A2:** **Areas of significant reduction of FA values for the comparison of patients with left-sided lesions versus normal controls**.

Fractional anisotropy decrease – FCD left-sided					
	JHU		MNI	*X, Y, Z*	*p*-Value
3%	Forceps minor	3%	Frontal lobe	91, 143, 91	0.014
16%	Anterior thalamic radiation	5%	Frontal lobe	69, 169, 91	0.022
3%	R fronto-occiptal fasciculus	5%	Frontal lobe	69, 169, 91	0.022
8%	R anterior thalamic radiation	1%	Frontal lobe	57, 158, 91	0.022
45%	R anterior thalamic radiation	20%	Caudate	68, 138, 91	0.036
3%	R corticospinal tract	–	Unidentified	65, 122, 91	0.022
3%	L corticospinal tract	–	Unidentified	65, 122, 91	0.022
3%	Inferior R fronto-occiptal fasciculus	–	Unidentified	63, 97, 91	0.022
16%	L cingulum	1%	Frontal lobe	106, 160, 91	0.014
3%	L fasciculus uncinatus	1%	Frontal lobe	111, 168, 91	0.036
3%	Inferior L fronto-occiptal fasciculus	1%	Frontal lobe	111, 168, 91	0.036
45%	L anterior thalamic radiation	13%	Caudate	111, 136, 91	0.014
18%	L superior longitudinal fasciculus (temporal part)	1%	Parietal lobe	130, 80, 91	0.014
3%	L superior longitudinal fasciculus	2%	Occipital lobe	119, 65, 91	0.034
11%	Inferior L fronto-occipital fasciculus	1%	Occipital lobe	120, 65, 91	0.034
5%	L inferior longitudinal fasciculus	1%	Occipital lobe	120, 65, 91	0.034
5%	L forceps major	2%	Occipital lobe	119, 65, 91	0.034
8%	Inferior L fronto-occipital fasciculus	2%	Occipital lobe	119, 65, 91	0.034
18%	L superior longitudinal fasciculus	1%	Parietal lobe	130, 80, 91	0.034

*JHU, Areas according to the JHU white matter atlas; MNI, Areas according to the MNI coordinates*.

**Table A3:** **Areas of significant reduction of FA values for the comparison of all patients after flipping the images with right-sided lesions to the left side versus normal controls**.

Fractional anisotropy decrease – patients group flipping the images with right-sided lesions					
	JHU		MNI	*X, Y, Z*	*p*-Value
3%	Inferior R fronto-occipital fasciculus	17%	Frontal lobe	60, 165, 91	0.038
3%	R superior longitudinal fasciculus	9%	Frontal lobe	59, 164, 91	0.044
3%	R anterior thalamic radiation	18%	Frontal lobe	62, 165, 91	0.068
3%	L corticospinal tract	1%	Caudate	67, 122, 91	0.042
8%	R anterior thalamic radiation	13%	Caudate	69, 122, 91	0.042
3%	R corticospinal tract	–	Unidentified	64, 119, 91	0.046
3%	Forceps minor	8%	Frontal lobe	95, 144, 91	0.014
8%	L cingulum	–	Unidentified	100, 148, 91	0.014
3%	L fasciculus uncinatus	1%	Frontal lobe	112, 167, 91	0.026
3%	Inferior L fronto-occipital fasciculus	3%	Frontal lobe	107, 172, 91	0.026
37%	L anterior thalamic radiation	5%	Caudate	111, 141, 91	0.022
5%	L superior longitudinal fasciculus	17%	Frontal lobe	137, 127, 91	0.038
5%	L superior longitudinal fasciculus (temporal part)	17%	Frontal lobe	137, 127, 91	0.038
11%	Forceps major	2%	Parietal lobe	114, 71, 91	0.086
3%	L Cingulum (hippocampus)	1%	Parietal lobe	115, 70, 91	0.048
11%	L inferior longitudinal fasciculus	53%	Occipital lobe	120, 68, 91	0.022
5%	Forceps major	29%	Occipital lobe	115, 54, 91	0.038
3%	Inferior L fronto-occipital fasciculus	1%	Parietal lobe	115, 54, 91	0.038
5%	Inferior L fronto-occipital fasciculus	53%	Occipital lobe	124, 46, 91	0.032
16%	L superior longitudinal fasciculus	2%	Parietal lobe	129, 84, 91	0.018
13%	L superior longitudinal fasciculus (temporal part)	2%	Parietal lobe	129, 84, 91	0.018
3%	L superior longitudinal fasciculus	6%	Temporal lobe	135, 83, 91	0.022
3%	L inferior longitudinal fasciculus	54%	Occipital lobe	125, 49, 91	0.032

*JHU, Areas according to the JHU white matter atlas; MNI, Areas according to the MNI coordinates*.
